# Refractory Hypophosphatemia Secondary to Tumor-Induced Osteomalacia: Diagnostic Challenges and Successful Management With Burosumab

**DOI:** 10.7759/cureus.90016

**Published:** 2025-08-13

**Authors:** Amna Ramzan, Fahad Aftab Khan Lodhi, Fatima Ramzan

**Affiliations:** 1 Family Medicine, Louisiana State University Health Sciences Center, Baton Rouge, USA; 2 Nephrology, Lake Charles Memorial Hospital/The Kidney Clinic, Lake Charles, USA; 3 Internal Medicine, Rashid Latif Medical College, Lahore, PAK

**Keywords:** burosumab, fibroblast growth factor-23, hypophosphatemia, phosphate homeostasis, tumor-induced osteomalacia

## Abstract

Tumor-induced osteomalacia (TIO) is a rare paraneoplastic syndrome characterized by fibroblast growth factor-23 (FGF-23) overproduction from mesenchymal tumors, leading to renal phosphate wasting, hypophosphatemia, and impaired 1,25-dihydroxyvitamin D (1,25 Vit D) synthesis. This report details a 69-year-old woman with obesity post-gastric bypass, iron deficiency anemia treated with ferric carboxymaltose (FCM) six months prior, osteoporosis post-zoledronic acid, hiatal hernia, and gastroesophageal reflux disease. She presented with generalized weakness, fatigue, and severe refractory hypophosphatemia (0.9 mg/dL) unresponsive to high-dose sodium phosphate supplementation. Laboratory evaluation showed renal phosphate loss (fractional excretion of phosphate (FEPO4) 10.4%, 24-hour urine phosphate > 100 mg/day), normal 25-hydroxyvitamin D (42 ng/mL), low-normal 1,25 Vit D (18 pg/mL), elevated parathyroid hormone (PTH) (110 pg/mL), and initially normal C-terminal FGF-23 (53 RU/mL). Genetic testing via the Renasight panel was negative for pathogenic mutations, including those associated with X-linked hypophosphatemia (XLH), and whole-exome sequencing revealed no CYP27B1 mutations. Repeat testing with an intact FGF-23 assay demonstrated elevation (92 pg/mL), supporting a diagnosis of TIO despite the remote FCM exposure and negative NetSpot scan (Etwok Inc., Dover, DE, United States) for tumor localization. Burosumab (0.5 mg/kg monthly) was initiated, normalizing serum phosphate without additional supplementation, though side effects included headaches and dental caries. This case highlights the critical role of intact FGF-23 assays over C-terminal assays for improved diagnostic accuracy in TIO, the indicative value of hypophosphatemia with low or inappropriately normal 1,25 Vit D, the necessity of understanding phosphate homeostasis feedback loops, and the efficacy of burosumab therapy in cases of strong clinical suspicion despite elusive tumors. Ongoing annual imaging aims for potential curative resection.

## Introduction

Tumor-induced osteomalacia (TIO) is a rare paraneoplastic syndrome characterized by fibroblast growth factor-23 (FGF-23)-secreting mesenchymal tumors that cause renal phosphate wasting, hypophosphatemia, and osteomalacia, leading to debilitating musculoskeletal symptoms [1,2].

Diagnosing TIO presents significant challenges, as it must be differentiated from mimics such as genetic hypophosphatemic disorders or iatrogenic causes like ferric carboxymaltose (FCM)-induced hypophosphatemia [3]. Comorbidities, including gastric bypass surgery, can exacerbate phosphate malabsorption in up to 30% of affected individuals, while FCM administration triggers transient FGF-23 elevation in 50%-92% of cases, typically resolving within 3-6 months; prolonged symptoms beyond this period should raise suspicion for FGF-23-mediated hypophosphatemia, which includes both acquired (e.g., TIO) and genetic causes [4-6]. A key diagnostic hallmark is hypophosphatemia accompanied by low or inappropriately normal 1,25-dihydroxyvitamin D (1,25 Vit D) levels, reflecting FGF-23's inhibition of renal 1-alpha-hydroxylase (CYP27B1) despite the stimulatory effects of low phosphate [7]. The choice of FGF-23 assay is critical: intact assays offer superior sensitivity (84%-90%) for detecting bioactive hormone, whereas C-terminal assays may yield false negatives by measuring inactive fragments [8]. Diagnostic delays often span 5-15 years, with 20%-40% of tumors remaining undetected despite imaging [9,10].

In such cases, curative tumor resection is ideal, but when localization fails, burosumab, a monoclonal antibody targeting FGF-23, restores phosphate homeostasis and 1,25 Vit D production, with dosing adjusted every four weeks based on serum phosphorus to avoid hyperphosphatemia and monitor side effects like headaches [11,12]. Hypophosphatemia, defined as serum phosphate below 2.5 mg/dL, arises from disrupted homeostasis involving intricate feedback among parathyroid hormone (PTH), vitamin D, and FGF-23, where low phosphate normally stimulates CYP27B1 to enhance absorption via increased 1,25-dihydroxyvitamin D, while PTH (stimulated by low calcium) provides additional regulation but is suppressed by hypophosphatemia, but elevated FGF-23 counters this by promoting phosphaturia and inhibiting 1,25 Vit D synthesis [13-15]. Normal 25-hydroxyvitamin D levels distinguish TIO from nutritional deficiencies, and secondary hyperparathyroidism may ensue from resultant hypocalcemia [13].

Genetic testing, such as the Renasight panel screening 397 genes including PHEX, FGF23, and CYP27B1, rules out hereditary mimics like X-linked hypophosphatemia (XLH) or vitamin D-dependent rickets, with negative results supporting acquired etiologies like TIO; whole-exome sequencing further excludes rare variants [16]. Advanced imaging with 68Ga-DOTATATE PET/CT (NetSpot; Etwok Inc., Dover, DE, United States) excels in tumor localization due to high somatostatin receptor affinity, surpassing alternatives like 18F-FDG PET/CT [17]. 

This case highlights refractory hypophosphatemia complicated by gastric bypass and prior FCM, underscoring the need for comprehensive evaluation to guide targeted therapy. Here, we describe the case of a 69-year-old woman who presented with persistent hypophosphatemia, renal phosphate wasting, and elevated intact FGF-23 levels despite a normal C-terminal assay, ultimately managed successfully with burosumab in the absence of tumor localization. This patient's journey illustrates the diagnostic pitfalls and therapeutic advancements in phosphaturic disorders, paving the way for the ensuing case presentation and in-depth discussion.

## Case presentation

A 69-year-old woman with a history of obesity status post-gastric bypass, iron deficiency anemia treated with FCM six months earlier, osteoporosis managed with zoledronic acid, hiatal hernia, and gastroesophageal reflux disease presented with progressive generalized weakness and fatigue over several weeks. Physical examination revealed muscle weakness without focal neurological deficits.

Laboratory findings, summarized in Table 1, included severe hypophosphatemia refractory to high-dose oral and intravenous sodium phosphate supplementation. Further workup demonstrated renal phosphate wasting, normal 25OHD, low-normal 1,25 Vit D, elevated PTH, and normal C-terminal FGF-23. Genetic evaluation via the Renasight panel excluded pathogenic mutations for XLH and related disorders, and whole-exome sequencing confirmed no CYP27B1 mutations, ruling out vitamin D-dependent rickets type 1A.

**Table 1:** Key laboratory findings

Test	Value	Reference range
Serum phosphate	0.9 mg/dL	2.5-4.5 mg/dL
Fractional excretion of phosphate (FEPO4)	10.40%	<5%
24-hour urine phosphate	>100 mg/day	<100 mg/day (with hypophosphatemia)
25-hydroxyvitamin D (25OHD)	42 ng/mL	30-100 ng/mL
1,25-Dihydroxyvitamin D (1,25 Vit D)	18 pg/mL	18-72 pg/mL
Parathyroid hormone (PTH)	110 pg/mL	15-65 pg/mL

**Figure 1:**
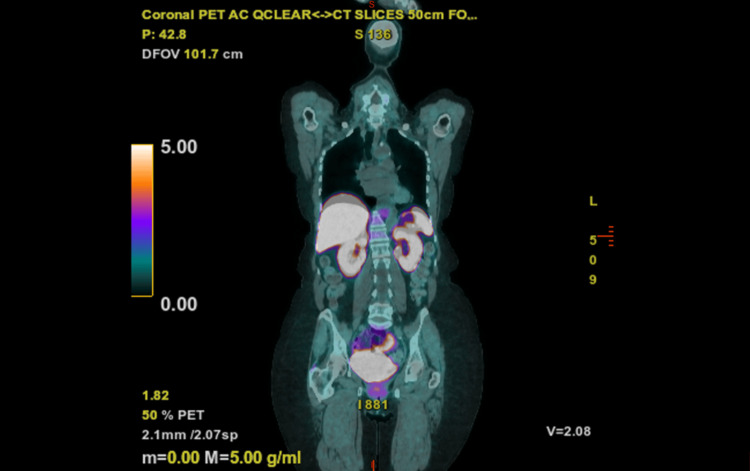
Representative image from 68Ga-DOTATATE PET/CT scan demonstrating no evidence of somatostatin receptor-positive tumors

Given the persistence beyond FCM's typical resolution window (>6 months), repeat FGF-23 testing using an intact assay revealed elevation, strongly suggesting TIO [4-6]. Imaging with 68Ga-DOTATATE PET/CT (NetSpot) was negative for somatostatin receptor-positive tumors (Figure 1) [9,10]. Burosumab was initiated at 0.5 mg/kg subcutaneously monthly, with dose adjustments based on pre-infusion phosphate levels. Serum phosphate normalized within weeks without further supplementation, improving symptoms. Side effects included mild headaches and dental caries, managed conservatively. Annual NetSpot imaging continues to seek a curative tumor resection [9,10].

## Discussion

This case exemplifies the diagnostic intricacies of refractory hypophosphatemia, where gastric bypass and prior FCM complicated the differential. FCM induces transient FGF-23 elevation in 50%-92% of cases, but symptoms resolve within 3-6 months; persistence beyond this favors TIO [4-6].

Genetic testing is essential to rule out hereditary phosphaturic disorders that mimic TIO [12,16]. The Renasight panel, which analyzes 397 genes associated with monogenic kidney diseases, includes several linked to hypophosphatemia and renal phosphate wasting. Key genes tested include PHEX (associated with X-linked hypophosphatemic rickets; mutations lead to increased FGF-23 levels and phosphate wasting), FGF-23 (autosomal dominant hypophosphatemic rickets; gain-of-function mutations stabilize FGF-23, inhibiting phosphate reabsorption), DMP1 and ENPP1 (autosomal recessive hypophosphatemic rickets types 1 and 2; mutations impair FGF-23 regulation, causing elevated FGF-23 and hypophosphatemia), SLC34A1 and SLC34A3 (hereditary hypophosphatemic rickets with hypercalciuria and Fanconi renotubular syndrome; encode sodium-phosphate cotransporters, with mutations reducing renal phosphate reabsorption), CLCN5 (Dent disease; impairs proximal tubular function, leading to phosphate wasting), and VDR (vitamin D-resistant rickets; disrupts vitamin D signaling, affecting phosphate homeostasis) [12,16]. Negative results, as in this patient, support an acquired etiology like TIO and guide management away from lifelong genetic counseling implications. Furthermore, given the initially normal C-terminal FGF-23 (which can be falsely normal due to detection of inactive fragments), and to comprehensively exclude rare acquired or genetic defects in vitamin D metabolism that could mimic TIO, such as VDR type 1A (VDDR1A), characterized by impaired 1-alpha-hydroxylation and low 1,25 Vit D, whole-exome sequencing was pursued specifically to screen for novel or unreported mutations in CYP27B1, the gene encoding 1-alpha-hydroxylase [16]. This step was crucial as standard panels might miss atypical variants, ensuring a thorough differential diagnosis before attributing symptoms to TIO.

Central to diagnosis is recognizing hypophosphatemia with low or inappropriately normal 1,25 Vit D as a hallmark of TIO, reflecting FGF-23's suppression of CYP27B1 despite hypophosphatemia's stimulatory effect [1,2,7]. It is also important to check 25OHD levels, as normal values (as seen here) help differentiate TIO from nutritional vitamin D deficiency, where low 25OHD would lead to secondary hypophosphatemia with appropriately low 1,25 Vit D but without the FGF-23-driven renal wasting profile [13,14]. Secondary hyperparathyroidism, as seen here (elevated PTH), arises from 1,25 Vit D deficiency, causing transient hypocalcemia [13].

Phosphate homeostasis comprehension is essential: Low PO4 stimulates CYP27B1 to boost 1,25 Vit D and PO4 absorption, while FGF-23 provides counter-regulation by inhibiting these pathways [13-15]. Disruptions, as in TIO, lead to unchecked phosphaturia [1,13]. Key interactions in phosphate homeostasis are summarized in Table 2. 

**Table 2:** Key interactions in phosphate homeostasis

Stimulus/condition	Effect
Low PO4	Stimulates CYP27B1 → increases 1,25 Vit D → enhances PO4 absorption/reabsorption
High PTH	Stimulates CYP27B1 and renal PO4 excretion
Low Ca	Stimulates PTH secretion
High FGF-23	Inhibits CYP27B1 and renal PO4 reabsorption
High PO4	Stimulates FGF-23 secretion
High 1,25 Vit D	Inhibits CYP27B1 (negative feedback)

FGF-23 assay choice is crucial: Intact assays measure bioactive FGF-23 with superior sensitivity (84%-90%) and correlation to outcomes in TIO, whereas C-terminal assays detect inactive fragments, potentially yielding false normals (as in this case) [2,7,8]. In this patient, the intact FGF-23 was measured using the MedFrontier ELISA assay, with a reference interval of 22 pg/mL that is approximately equivalent to the Kainos > 30 pg/mL used for XLH trial inclusion, while >68 pg/mL aligns with the Kainos > 100 pg/mL for TIO trials. These equivalents are derived from linear regression analyses in comparative studies, accounting for the MedFrontier assay's lower bias. In this case, the patient's intact FGF-23 level of 92 pg/mL exceeded the adjusted TIO cutoff by approximately 1.35-fold (mild elevation) and the XLH cutoff by over fourfold (moderate elevation), signifying pathological FGF-23 overproduction; this elevation, combined with persistent hypophosphatemia, renal phosphate wasting, inappropriately low-normal 1,25 Vit D, negative genetic testing, and rapid symptom resolution with burosumab (an FGF-23 inhibitor), compellingly supports an FGF-23-mediated phosphaturic disorder such as TIO, despite the elusive tumor on imaging. Diagnostic delays average 5-15 years, with 20%-40% of tumors undetected [3,7].

NetSpot (68Ga-DOTATATE PET/CT) remains superior to other modalities such as 18F-FDG PET/CT and 111In-octreotide scintigraphy for localizing phosphaturic mesenchymal tumors in TIO, owing to its higher affinity for somatostatin receptors and improved spatial resolution [17]. When tumors evade detection despite advanced imaging (e.g., NetSpot), burosumab, a human monoclonal antibody that binds to FGF-23 and inhibits its biological activity, thereby restoring renal phosphate reabsorption and increasing 1,25 Vit D production, is indicated for strong clinical suspicion [11,12]. Dosed every four weeks (annual cost ~$200,000-$400,000), dosing should be adjusted based on serum phosphorus levels during active therapy to maintain euphosphatemia while avoiding hyperphosphatemia, with regular monitoring to optimize efficacy and minimize side effects like headaches and dental issues [11,12]. Curative resection remains ideal [3,9,10]. The causes of hypophosphatemia can be categorized into renal and non-renal types, as summarized in Table 3 [13,16].

**Table 3:** Causes of hypophosphatemia (renal vs. non-renal)

Category	Description	Examples
Renal causes	Involve increased urinary phosphate excretion due to impaired tubular reabsorption, often with elevated fractional excretion of phosphate (>5%-10%). These are common in genetic or acquired tubular disorders and account for many chronic, refractory cases.	Primary hyperparathyroidism (PTH-mediated phosphaturia); Fanconi syndrome (generalized tubular dysfunction leading to phosphate, glucose, and amino acid wasting); tumor-induced osteomalacia (TIO; FGF-23-mediated inhibition of NaPi cotransporters); X-linked hypophosphatemia (XLH; PHEX mutations causing elevated FGF-23); autosomal dominant hypophosphatemic rickets (ADHR; FGF23 mutations); diuretic use (e.g., loop or thiazide diuretics interfering with reabsorption); osmotic diuresis (e.g., in uncontrolled diabetes); post-kidney transplant hypophosphatemia (due to tertiary hyperparathyroidism or FGF-23 elevation); renal tubular acidosis (impaired bicarbonate reabsorption affecting phosphate handling) [13,16].
Non-renal causes	Encompass decreased intestinal absorption or intracellular shifts without primary renal wasting. These are often acute and reversible, with normal or low fractional excretion of phosphate (<5%).	Decreased absorption: malnutrition/starvation (poor dietary intake); vitamin D deficiency (impaired mineralization and absorption); antacid overuse (aluminum/magnesium binding phosphate); gastric bypass surgery (malabsorption, as in this case); celiac disease or inflammatory bowel disease (impaired gut absorption). Internal shifts: refeeding syndrome (insulin-driven phosphate uptake into cells); respiratory alkalosis (hyperventilation causing intracellular shift); insulin therapy (e.g., in diabetic ketoacidosis); acute pancreatitis (saponification and sequestration); severe burns or trauma (cellular uptake during repair); hungry bone syndrome (post-parathyroidectomy bone remineralization); sepsis or acute illness (cytokine-mediated shifts) [13].

## Conclusions

This case underscores the importance of intact FGF-23 testing for accurate TIO diagnosis, the diagnostic clue of hypophosphatemia with low/inappropriately normal 1,25 Vit D, the value of phosphate homeostasis knowledge, and prompt burosumab initiation when tumors are elusive or detected but inoperable (e.g., due to location not amenable to surgery). Additionally, regular follow-up imaging is crucial to continue searching for mesenchymal tumors, as surgical resection offers a curative option by eliminating the source of FGF-23 overproduction. Early recognition and management can significantly enhance patient outcomes.
